# Examining the factorial validity of the Quality of Life Scale

**DOI:** 10.1186/s12955-020-01292-5

**Published:** 2020-02-18

**Authors:** Ashley J. Reeves, Russell T. Baker, Madeline P. Casanova, Scott W. Cheatham, Michael A. Pickering

**Affiliations:** 1 University of Idaho, 875 Perimeter Drive MS2401, Moscow, ID 83844 USA; 2 California State University Dominguez Hills, 1000 E. Victoria Street, Carson, California 90747 USA

**Keywords:** Exploratory factor analysis, Confirmatory factor analysis, Covariance modeling, Instrument development, Physically active, Healthcare, Well-being

## Abstract

**Background:**

Quality of life (QoL) is important to assess in patient care. Researchers have previously claimed validity of the Quality of Life Scale (QOLS) across multiple samples of individuals, but close inspection of results suggest further psychometric investigation of the instrument is warranted. Therefore, the purposes of this study were to: 1) evaluate the proposed five-factor, 15-item and three-factor, 16-item QOLS; 2) if the factor structure could not be confirmed, re-assess the QOLS using exploratory factor analysis (EFA) and covariance modeling to identify a parsimonious refinement of the QOLS structure for future investigation.

**Methods:**

Participants varying in age, physical activity level, and identified medical condition(s) were recruited from clinical sites and ResearchMatch. Confirmatory factor analyses (CFA) were performed on the full sample (*n* = 1036) based on proposed 15- and 16-item QOLS versions. Subsequent EFA and covariance modeling was performed on a random subset of the data (n_1_ = 518) to identify a more parsimonious version of the QOLS. The psychometric properties of the newly proposed model were confirmed in the remaining half of participants (n_2_ = 518). Further examination of the scale psychometric properties was completed using invariance testing procedures across sex and health status sub-categories.

**Results:**

Neither the 15- nor 16-item QOLS CFA met model fit recommendations. Subsequent EFA and covariance modeling analyses revealed a one-factor, five-item scale that satisfied contemporary statistical and model fit standards. Follow-up CFA confirmed the revised model structure; however, invariance testing requirements across sex and injury status subgroups were not met.

**Conclusions:**

Neither the 15- nor 16-item QOLS exhibited psychometric attributes that support construct validity. Our analyses indicate a new, short-form model, might offer a more appropriate and parsimonious scale from some of the original QOLS items; however, invariance testing across sex and injury status suggested the psychometric properties still vary between sub-groups. Given the scale design concerns and the results of this study, developing a new instrument, or identifying a different, better validated instrument to assess QoL in research and practice is recommended.

## Background

Assessing patient reported outcomes through a multidimensional lens (e.g., patient symptomatology, functional status, quality of life, etc.) is an important component of healthcare research and practice [1]. Quality of life (QoL), which may date back to Aristotle [2], is a longstanding and valued construct assessed in patient care and intervention research [3]. According to existing literature, QoL may include a variety of factors, including life satisfaction [4, 5], disease- or condition-specific symptoms [6], mood, and functional status [1, 7]. The multi-faceted concept of QoL, coupled with a lack of agreement on what it should entail, limit its usefulness in informing patient care decisions, despite its importance.

Inconsistently applied definitions of QoL, particularly in health care fields, make it difficult to accurately and consistently assess [1, 8]. For example, Gill and Feinstein (1994) examined 75 studies with 159 QoL instruments and identified a lack of coherence in meaning between many of the instruments [9]. Along with a lack of clarity on a definition, the notion that ill or injured individuals perceive QoL differently than healthy individuals adds to the confusion. This belief, however, is not well supported in the literature [1, 7, 8, 10]. Individuals, regardless of health or injury status, recognize and respond to the same QoL factors; however, the relative importance of these factors (e.g., functional impairments) can vary across the lifespan or by specific situations [1, 7]. Therefore, when assessing the effectiveness of provided patient care services, healthcare providers should recognize that physical health status is only one of the factors affecting an individual’s overall QoL [1, 8].

Given the lack of clarity, there is a need for QoL scales to be consistent and meaningful to most individuals [8]. Instruments should be psychometrically sound and assess appropriate dimensions of QoL without blending with other related, but distinct health constructs (e.g., functional performance) [1]. One commonly used instrument is the Flanagan Quality of Life Scale (QOLS). The original QOLS consisted of fifteen items and was intended to measure five different aspects (i.e. “factors”) of QoL: 1) physical and material well-being (PMWB), 2) relations with other people (REL), 3) social, community, and civic activities (SCC), 4) personal development and fulfillment (PDF), and 5) recreation (REC) [8]. A modified version of the QOLS was developed for use with chronically ill patients (e.g., fibromyalgia, cardiac disease, arthritis, posttraumatic stress disorder, diabetes, etc.), by adding a sixteenth item to assess independence. The 16-item version is more commonly used than the 15-item version [8] and aimed to assess three distinct factors of QoL: 1) relationships and material well-being (RMW), 2) personal, social, and community commitment (PSCC), and 3) health and functioning (HF) [10]. For both versions of the QOLS, individuals score items using a 1 (“terrible”) to 7 (“delighted”) point Likert scale. The QOLS has been studied in healthy populations, chronic illness groups, and adults of all ages [8, 10–15]. It has not, however, been studied in children, and therefore, is not currently recommended for use in youth populations [8].

Although the QOLS has been suggested to be a reliable and valid scale [10–15], psychometric findings have been inconsistent, and frequently fail to meet recommended guidelines for establishing scale validity [16, 17] (Tables 1 & 2). In addition, across multiple studies with diverse samples, published factor structures have varied [10–15] (Tables 1 & 2) and do not meet recommended guidelines [16, 18]. For example, findings in most studies of the original 15-item version are inconsistent with the originally proposed five-factor structure [10–15], which indicates the theoretical framework of the scale is not well-supported. Similarly, studies using the 16-item QOLS have found that items typically factor into three dimensions [10–15], however, the specific factor make-up (e.g., using the same items within dimensions), has varied (Tables 1 & 2). Studies have also attempted to assess internal consistency, test-retest reliability, validity of the scale presented in different languages, and concurrent validity with other instruments [10–15], but these results must be interpreted carefully due to the lack of a consistent factor structure. Thus, further investigation of the psychometric properties of the scale is warranted.

**Table 1:** Exploratory factor analysis solutions for the QOLS items from Burckhardt, et al. (2003)^10^

Subjects	Sample 1	Sample 2	Sample 3	Sample 4	Sample 5
**Item**^**10**^	**Factor**	**Factor**	**Factor**	**Factor**	**Factor**
1. “Material comforts home, food, conveniences, financial security…”	HF	RMW*	RMW	*DNF*	RMW
2. “Health – being physically fit and vigorous…”	HF	HF	HF	HF	HF
3. “Relationships with parents, siblings & other relatives – communicating, visiting, helping…”	RMW*	RMW	PSCC#	RMW	RMW
4. “Having and raising children…”	RMW	RMW	RMW*	RMW	RMW
5. “Relationships with spouse or significant other…”	RMW*	RMW	RMW	RMW	RMW
6. “Close friends…”	RMW	RMW*	RMW*	RMW	RMW*
7. “Helping and encouraging others, volunteering, giving advice…”	PSCC#	PSCC	PSCC*	PSCC	PSCC
8. “Participating in organizations and public affairs…”	PSCC	PSCC	PSCC	PSCC	PSCC
9. “Learning – attending school, improving understanding, getting additional knowledge…”	PSCC	PSCC	PSCC	PSCC	PSCC
10. “Understanding yourself – knowing your assets and limitations – knowing what life is about…”	PSCC$	PSCC	PSCC	PSCC	PSCC
11. “Work – job or in home…”	HF	HF	HF	HF	HF*
12. “Expressing yourself creatively…”	PSCC	PSCC*	PSCC*	PSCC	PSCC
13. “Socializing – meeting other people, doing things, parties, etc.”	RMW	PSCC*	PSCC*	HF#	PSCC$
14. “Reading, listening to music, or observing entertainment…”	RMW*	PSCC	PSCC	*DNF*	PSCC
15. “Participating in active recreation…”	HF	HF	HF	HF	HF
16. “Independence, doing for yourself…”	*NI*	*NI*	*NI*	HF	HF

*RMW* Relationships and Material Well-being; *PSCC* Personal, Social, and Community Commitment; *HF* Health and Functioning; *NI* Item Not Included in Analysis; *DNF* Item Did Not Factor; * = Item had substantial cross-loading (i.e., ≥ .30, but ≤ .44) on another factor; # = Item had extreme cross-loading (i.e., ≥ .45) on another factor. $ = Item had substantial cross-loadings (i.e., ≥ .30) on two other factors. Sample 1 = 319 healthy Swedish and American individuals; Sample 2 = 584 Americans with chronic illness; Sample 3 = 170 Swedish women with chronic rheumatic disease; Sample 4 = 353 men; Sample 5 = 888 women. Each item is labeled in the proposed construct from that paper

**Table 2:** Exploratory factor analysis solutions across studies validating the Flanagan Quality of Life Scale

Subjects	Sample 1	Sample 2	Sample 3
**Item**	**Factor**	**Factor**	**Factor**
1. Material comforts home, food, conveniences, financial security…	RMW	PWSB*	MWBPD*
2. Health – being physically fit and vigorous…	HF	AP	PSCC*
3. Relationships with parents, siblings & other relatives – communicating, visiting, helping…	RMW	RO	IR
4. Having and raising children…	RMW	RO	IR
5. Relationships with spouse or significant other…	RMW	RO	IR*
6. Close friends…	RMW	RO	IR
7. Helping and encouraging others, volunteering, giving advice…	PSCC	PWSB*	IR
8. Participating in organizations and public affairs…	PSCC	PWSB	PSCC*
9. Learning – attending school, improving understanding, getting additional knowledge…	PSCC	PWSB	PSCC
10. Understanding yourself – knowing your assets and limitations – knowing what life is about…	PSCC!	PWSB	MWBPD
11. Work – job or in home…	HF	AP*	MWBPD
12. Expressing yourself creatively…	PSCC	PWSB	MWBPD
13. Socializing – meeting other people, doing things, parties, etc.….	PSCC	PWSB$	PSCC*
14. Reading, listening to music, or observing entertainment…	PSCC	PWSB$	PSCC*
15. Participating in active recreation…	HF	AP	PSCC*
16. Independence, doing for yourself…	HF	AP	MWBPD#

Factor Names: *RMW* Relationships and Material Well-being; *PSCC* Personal, Social, and Community Commitment; *HF* Health and Functioning; *PSWB* Personal and Social Well-being; *RO* Relationship with Others; *AP* Active Participation; *MWBPD* Material Well-being and Personal Development; *IR* Interpersonal relationships. *Other Abbreviations and Symbols: NI* Item Not Included in Analysis; *DNF* Item Did Not Factor; * = Item had substantial cross-loading (i.e., ≥ .30, but ≤ .44) on another factor; # = Item had extreme cross-loading (i.e., ≥ .45) on another factor. $ = Item had substantial cross-loadings (i.e., ≥ .30) on two other factors; ^ = authors did not provide cross-loading values;! = Item in factor but loading was less than .35. Sample 1 = 140 Spanish women with fibromyalgia(15); Sample 2= 113 Swedish women with fibromyalgia(12); Sample 3 = 146 German patients with fibromyalgia(14)

In short, factorial validity and consistency of the scale across populations is not well-supported [10–15] (Tables 1 & 2). Further, at least three additional steps beyond EFA are necessary to establish that a version of the QOLS is sound for use in practice and research. These include: 1) EFA re-analysis to identify items with a more consistent factor structure, 2) confirmatory factor analysis (CFA) to more rigorously examine the structure and, 3) CFA-based invariance testing to explore measurement properties of the scale across subgroups of the population (e.g., gender, age, disease types, etc.) [16, 17]. Failure to establish equivalent measurement properties across groups risks introducing measurement bias, which confounds interpretation between group comparisons [16, 18].

A systematic CFA approach, subsequent to identifying a meaningful factor structure via EFA, offers a more complete and rigorous psychometric examination of an instrument’s measurement properties. Completing an invariance analysis facilitates logical refinement and stricter testing of its measurement properties [17–19]. Invariance testing of the QOLS would ensure that the operationalization of the construct ‘quality of life’ has the same meaning across groups. Ultimately, through this process, a more psychometrically sound instrument can be identified [16, 18]. Currently, psychometric analysis involving EFA refinement, followed by CFA and invariance testing, has not been conducted on the QOLS. Additionally, the scale has not yet been assessed in a group of participants defined as “physically active,” or across participants who are suffering from various stages (i.e., acute, sub-acute, and chronic) of musculoskeletal injury.

Despite the scale being used for over 40 years, the incomplete psychometric analysis of the QOLS is insufficient to justify widespread use. Therefore, the purposes of this study were to: 1) assess the factorial validity of the five-factor, 15-item and the three-factor, 16-item QOLS, and if these scales met model fit recommendations, 2) to assess measurement (i.e., equal forms, loadings, and intercepts) and structural (i.e., equal factor variances/ covariances, and equal means) invariance of the QOLS across gender and physical health status (i.e., physically active-healthy, physically active-injured, musculoskeletal pathology with a comorbidity, and osteoarthritis). A secondary purpose, if model fit did not hold or invariance testing could not be completed, was to: 1) re-examine the factor structure of the QOLS using an EFA and covariance modeling approach to identify a more parsimonious version of the QOLS for future investigation, 2) assess the newly proposed covariance QOLS model using CFA procedures, and if the new model met fit recommendations, 3) assess measurement and structural invariance of the revised QOLs across gender and health status.

## Methods

The present study was approved by University Institutional Review Board (IRB). Informed consent was obtained from all participants before data collection. Data were collected over the course of one year from various settings across the nation. Confidentiality of participant responses was ensured per the approved IRB protocol, and all data were deidentified prior to analysis.

### Participants

Adult participants were recruited from several locations across the nation to obtain a large heterogeneous sample that included different ages, physical activity levels, and medical conditions. Individuals were either recruited from: 1) athletic training clinics (*n* = 22), 2) outpatient rehabilitation clinics (n = 2; i.e., physically active individuals), or 3) ResearchMatch (*n* = 316; Vanderbilt University, Nashville, TN), a nationwide online database of research volunteers. Individuals who were physically active and classified as healthy or having an acute, sub-acute, or persistent injury were included in the study (Table 3). Individuals with chronic pain were excluded from the study as chronic pain has unpredictable patterns [20, 21]. Volunteers registered on ResearchMatch provide information about their health status and other pieces of personal or demographic information and are then randomly selected based on study criteria. For the present study, individuals recruited through ResearchMatch, were eligible to participate if they had either: 1) a musculoskeletal pathology with a comorbidity, or 2) osteoarthritis. Data from ResearchMatch contained identifiers to allow the survey to be email to participants, but the collected data were de-identified prior to analysis and all files containing respondent identifying information were deleted.

**Table 3:** Study definitions and terminology

Terminology	Definition
Physically Active^20^	“An individual who engages in athletic, recreational, or occupational activities that require physical skills and who uses strength, power, endurance, speed, flexibility, range of motion, or agility at least 3 days per week.”
**Injury Classification**^**20**^	
Healthy	“Free from musculoskeletal injury and fully able to participate in sport or activity.”
Acute Injury	“A musculoskeletal injury that precludes full participation in sport or activity for at least 2 consecutive days (0–72 h post-injury).”
Subacute Injury	“A musculoskeletal injury that precludes full participation in sport or activity for at least 2 consecutive days (3 days to 1-month post-injury).”
Persistent Injury	“A musculoskeletal injury that has been symptomatic for at least 1 month.”
Chronic Injury	“Pain that consistently does not get any better with routine treatment or non-narcotic medication.”
**Activity Level Classification**^**22**^	
Inactive	“No activity beyond baseline activity.”
Low	“Activity beyond baseline, but fewer than 150 min of moderate intensity exercise per week.”
Medium	“150–300 min of moderate intensity activity per week”.
High	“More than 300 min of moderate intensity activity per week.”
**Athlete Level**^**20**^	
Competitive Athlete	“A participant who engages in a sport activity that requires at least 1 pre-participation examination, regular attendance at scheduled practices and/or conditioning sessions and a coach who leads practices and/or competitions.”
Recreational Athlete	“Participants who meet the criteria for physical activity and participate in sport, but do not meet the criteria for competitive status.”
Occupational Athlete	“Participants who meet the criteria for physical activity for occupation or recreation, but do not meet the criteria for competitive or recreational athlete.”
Physically Active in ADLs	“Participants who do not meet the criteria for any “athlete” category, but who are physically active through their daily activities (e.g., physically active for at least 30 min per day, 3 days per week).”

*ADL* Activities of daily living. Definitions for physically active, injury classifications, and athlete level are from Vela and Denegar (2010).^20^ Activity level definitions are from the US Department of Health and Human Services^22^

From the total sample, individuals were also split into four different subgroups: 1) physically active healthy (PA-H), 2) physically active injured (PA-I), 3) musculoskeletal pathology with a comorbidity (MSK-C), and 4) osteoarthritis (OA). These subgroups were chosen to facilitate comparison across studies based on previous literature assessing factor structure of the QOLS [10]. Individuals in the PA-H and PA-I groups were classified based on a priori definitions used in previous literature (Table 3) [20]. Classifications included injury category (i.e., acute, subacute, persistent) and type of athlete (i.e., competitive, recreational, occupational, or physically active in activities of daily living [ADL]; Table 3) [20]. Individuals in all groups were also classified into one of four possible “activity levels” (i.e., inactive, low, medium, high; Table 3), as defined by the US Department of Health and Human Services [22].

### Instrumentation

A survey was created in paper and electronic form. The electronic survey was created using Qualtrics online software (Qualtrics, LLC, Provo, UT), with all paper responses also being input into Qualtrics for data analysis. Information collected was identical in both versions of the survey, and included basic demographics (e.g., age, sex, physical activity level, etc.) and the QOLS.

### Quality of Life Scale

The QOLS is an instrument created based on commonly identified factors that may pertain to QoL [8]. Both a 15- and 16-item version exist and have been studied in various populations [10–15]. The 16-item version includes all items in the 15-item version and the addition of one item aimed at evaluating independence as it pertains to one’s QoL [10]. Participants responded to the 16-item QOLS using a 7-point Likert scale, with 1 representing “terrible” and 7 representing “delighted” [8]. Item scores are summed together, with lower scores indicating poorer quality of life and higher scores indicating better quality of life [8].

### Data analysis

Data was initially analyzed using CFA maximum likelihood estimation procedures for both the 15- and 16-item QOLS. Because model fit did not meet recommended guidelines as outlined in the literature [16, 17], the data was then split randomly into two halves (n_1_, n_2_) with 518 participants in each sample. An EFA was conducted using the n_1_ sample to identify a more parsimonious and psychometrically sound solution. The n_1_ sample was also used to test the model using a more rigorous covariance model approach based on the final EFA solution. The covariance model was then confirmed using CFA with sample n_2_. Lastly, invariance testing using the full sample (i.e., n_1_ and n_2_ combined) was conducted to assess measurement and structural invariance of the QOLs across gender (i.e., male, female) and health status (i.e., PA-H, PA-I, OA). Finally, a covariance model latent variable correlation analysis and a composite score bivariate correlational analysis were conducted to determine if the modified version of the scale explained an acceptable percentage of the variance in responses on the original QOLS.

### Data cleaning

Data was exported from Qualtrics, and all analyses were conducted in Statistical Package for Social Sciences Version 24.0 (IBM Corp., Armonk, NY). Data was treated conservatively, and any participants missing more than 10% of the responses on the QOLS (i.e., 2 or more missing responses) were excluded from analysis. Remaining missing data was replaced with the rounded mean score of the respective item for analysis purposes. Participants with missing demographic data were not excluded from analysis. Data was assessed for normality using histograms, z-scores, and skewness and kurtosis values. Possible multivariate outliers were also identified using Malahanobis distance, for which the cut-off value for 16 degrees of freedom at a *p*-value of .001 was 39.252 [17].

### Confirmatory factor analysis of the 15- and 16-item Quality of Life Scale

The full sample was used to conduct a CFA using maximum likelihood estimation in Analysis of Moment Structures (AMOS) software (IBM Corp., Armonk, NY) on both the 15-item and 16-item scales. Responses for the original fifteen items were pulled from the full data set of sixteen items to examine the five-factor structure. Subsequently, the proposed three-factor, 16-item version was assessed using responses to all sixteen items. In order to assess correlations between the five-factor and three-factor latent constructs, additional first-order CFA’s were conducted on the 15 and 16-item QOLS. Model fit indices were evaluated based on a priori values to evaluate the originally proposed factor structures. The relative goodness-of-fit indices computed were the Comparative Fit Index (CFI; ≥ .95), Tucker-Lewis Index (TLI; ≥ .95), Root Mean Square Error of Approximation (RMSEA ≤ .06), and Bollen’s Incremental Fit Index (IFI; ≥ .95) [16, 17, 23]. The likelihood ratio statistic (Chi square or CMIN) was also assessed, but because it is heavily influenced by sample size, it was not used as the primary assessment of model fit [17, 19]. If model fit criteria were met, invariance testing was to be applied to the sample. Since model fit criteria were not met, EFA, covariance modeling, CFA, and invariance procedures were conducted to assess for a more valid revised factor structure.

### Identification of a modified Quality of Life Scale

The full sample was randomly split in half (i.e., Samples n_1_ and n_2_). Sample n_1_ was re-analyzed using EFA. EFA was conducted using maximum likelihood extraction; Bartlett’s test for sphericity and KMO for sampling adequacy were both assessed for violations. Cut-off values were set a priori at <.001 for Bartlett’s test of sphericity and ≥.80 for KMO, which are conservative compared to widely accepted values (KMO >.70, Bartlett’s <.05) [18]. Items with loadings less than .40 were removed, followed by items that cross-loaded on multiple factors at .30 or greater [18]. Items with loadings less than .30 were classified as “Did Not Factor” (DNF), and those with loadings less than .40 were classified as “Did Not Load” (DNL). For analysis purposes, cross loadings were defined as substantial (≥ .30 ≤ .44) or extreme (≥ .45).

Bivariate correlations between items, Cronbach’s alpha, and the concept each item was intended to measure were used to make removal decisions. Both Cronbach’s alpha and omega were used to estimate internal consistency [18, 24]. Cronbach’s alpha was set a priori as ≥ .70 and ≤ .89 [18]. Items were removed one at a time, and the EFA and Cronbach’s alpha were re-run after the removal of each item. This process continued until a parsimonious factor structure that met recommended statistical guidelines was met.

### Validation analysis of the modified Quality of Life Scale

The modified QOL scale identified during the EFA process was then re-assessed based on a more restricted covariance modeling specifying no cross loadings, using sample n_1_. The same criteria utilized for the initial CFA were used to assess model fit [17, 19]. The model was then confirmed via CFA using sample n_2_. Following confirmation of the new model invariance testing with the full sample was conducted to assess measurement and structural invariance of the modified QOLS across sex (i.e., male, female) and health status (i.e., physically active-healthy, physically active-injured, and osteoarthritis). Invariance testing ensures that across groups, factors (e.g., relationships and material well-being, personal, social, and community commitment, etc.) have identical items, the meaning of those factors are similar, and that the means of the factors can be meaningfully compared [17, 19]. Invariance was evaluated based on a CFI difference (CFI_DIFF_) of less than .01, and the chi-square difference test (χ^2^_DIFF_), with a *p*-value cut-off of 0.01 [17, 19]. Given the sensitivity of the χ^2^_DIFF_ test to sample size, the CFI_DIFF_ test held greater weight in decisions regarding invariance testing model fit.

### Correlation analyses

The total sample was used to assess the relationship between participant scores on the 16-item QOLS and the newly proposed modified QOLS. A covariance modeling approach was used to assess correlations using latent variable scores. Additionally, a bivariate correlation analysis was conducted using the cumulative scores from the 16-item scale and the cumulative scores on the newly proposed QOLS. An acceptable percentage of the variance explained was set at r ≥ 0.90 (R^2^ = 0.81) [25].

## Results

### Data cleaning & sample characteristics

A total of 1098 individuals completed the QOLS. In the sample, 64 (6.1%) individuals were missing a response to one item; the items with missing responses were replaced with the rounded mean of the respective item. Of the 1098 individuals with one or fewer missing responses on the QOLS, a total of 57 participants (5.2%) were identified as possible multivariate outliers and were removed from the final analysis. Five additional participants, who were part of the PA-H and PA-I subgroups, were excluded because injury category was not specified, and therefore, could not be classified into either the healthy or injured group. This left a total of 1036 individuals, ages 18–74 years old, in the final analysis for the full sample. The full sample was broken down into the following subgroups: PA-H (*n* = 151, 18–61 y), PA-I (*n* = 470, 18–74 y), MSK-C (*n* = 279, 19–65 y), and OA (*n* = 127, 27–65 y). Demographic information for the full sample and each subgroup is provided in Table 4.

**Table 4:** Demographic information across samples

	Full Sample (*n* = 1036)	Physically Active – Healthy (*n* = 151)	Physically Active – Injured (*n* = 470)	Musculoskeletal Pain & Comorbidity (*n* = 284)	Osteoarthritis (*n* = 131)
**Sex**					
Male	387 (37.4)	82 (54.3)	240 (51.1)	44 (15.5)	21 (16.0)
Female	641 (61.9)	69 (45.7)	229 (48.7)	236 (83.1)	107 (81.7)
Other	3 (0.3)	0 (0.0)	0 (0.0)	2 (0.7)	1 (0.8)
Unknown	5 (0.5)	0 (0.0)	1 (0.2)	2 (0.7)	2 (1.5)
**Age (years)**	33.87 ± 15.73	24.28 ± 8.18	24.28 ± 9.48	46.07 ± 12.10	53.93 ± 9.37
**Activity Level**^**21**^					
Inactive	142 (13.7)	3 (2.0)	31 (6.6)	81 (28.5)	27(20.6)
Low	294 (28.4)	16 (10.6)	89 (18.9)	126 (44.4)	63(48.1)
Medium	291 (28.1)	53 (35.1)	146 (31.1)	61 (21.5)	31(23.7)
High	302 (29.2)	78 (51.7)	203 (43.2)	13 (4.6)	8(6.1)
Unknown	7 (0.7)	1 (0.7)	1 (0.2)	3 (1.1)	2 (1.5)

Data is presented as number of individuals (% of total in sample). Age is given as mean ± SD. Activity levels are based on definitions of the US Department of Health and Human Services^21^

### Physically active healthy and physically active injured

Beyond the demographic information provided in Table 4, individuals in the physically active groups were also classified by level of competition within their respective sport based on definitions used in previous literature (Table 3) [20]. Individuals participated in a variety of sports and activities, adding to the heterogeneity of the sample. In the injured group, the most common sports or activities were soccer (*n* = 50, 10.6%), basketball (*n* = 48, 10.2%), and track and field (*n* = 47, 10.0%). In the healthy group, soccer (*n* = 17, 11.3%) and football (*n* = 13, 8.6%) were the most common. Information on classification and sport participation are presented in Table 5 [20]. Further classification of the injured individuals revealed that 217 (49.2%) had a persistent injury, 124 (26.4%) had an acute injury, and 129 (27.4%) had a subacute injury based on the definitions provided in Table 3 [20].

**Table 5:** Classification of physically active individuals

	Physically Active – Healthy (*n* = 151)	Physically Active – Injured (*n* = 470)
**Athlete Type**^**20**^		
Competitive	75 (49.7)	290 (61.7)
Recreational	34 (22.5)	63 (13.4)
Occupational	11 (7.3)	55 (11.7)
Activities of Daily Living	25 (16.6)	61 (13.0)
Unknown	6 (4.0)	1 (0.2)
**Sport**		
Baseball	6 (4.0)	33 (7.0)
Basketball	10 (6.6)	48 (10.2)
Cheerleading	1 (0.7)	7 (1.5)
Cross Country	2 (1.3)	3 (0.6)
Football	13 (8.6)	32 (6.8)
Racquet Sports	3 (2.0)	5 (1.1)
Recreational Running	9 (6.0)	7 (1.5)
Soccer	17 (11.3)	50 (10.6)
Softball	8 (5.3)	41 (8.7)
Swim and Dive	1 (0.7)	9 (1.9)
Track and Field	6 (4.0)	47 (10.0)
Volleyball	3 (2.0)	12 (2.6)
Weight Lifting	12 (7.9)	22 (4.7)
Other	29 (19.2)	82 (17.4)
No Sport Participation	24 (15.9)	70 (14.9)
Unknown	7 (4.6)	2 (0.4)

Data given as number of individuals (% of total in sample)

### Confirmatory factor analysis five-factor 15-item Quality of Life Scale

**Fig. 1:**
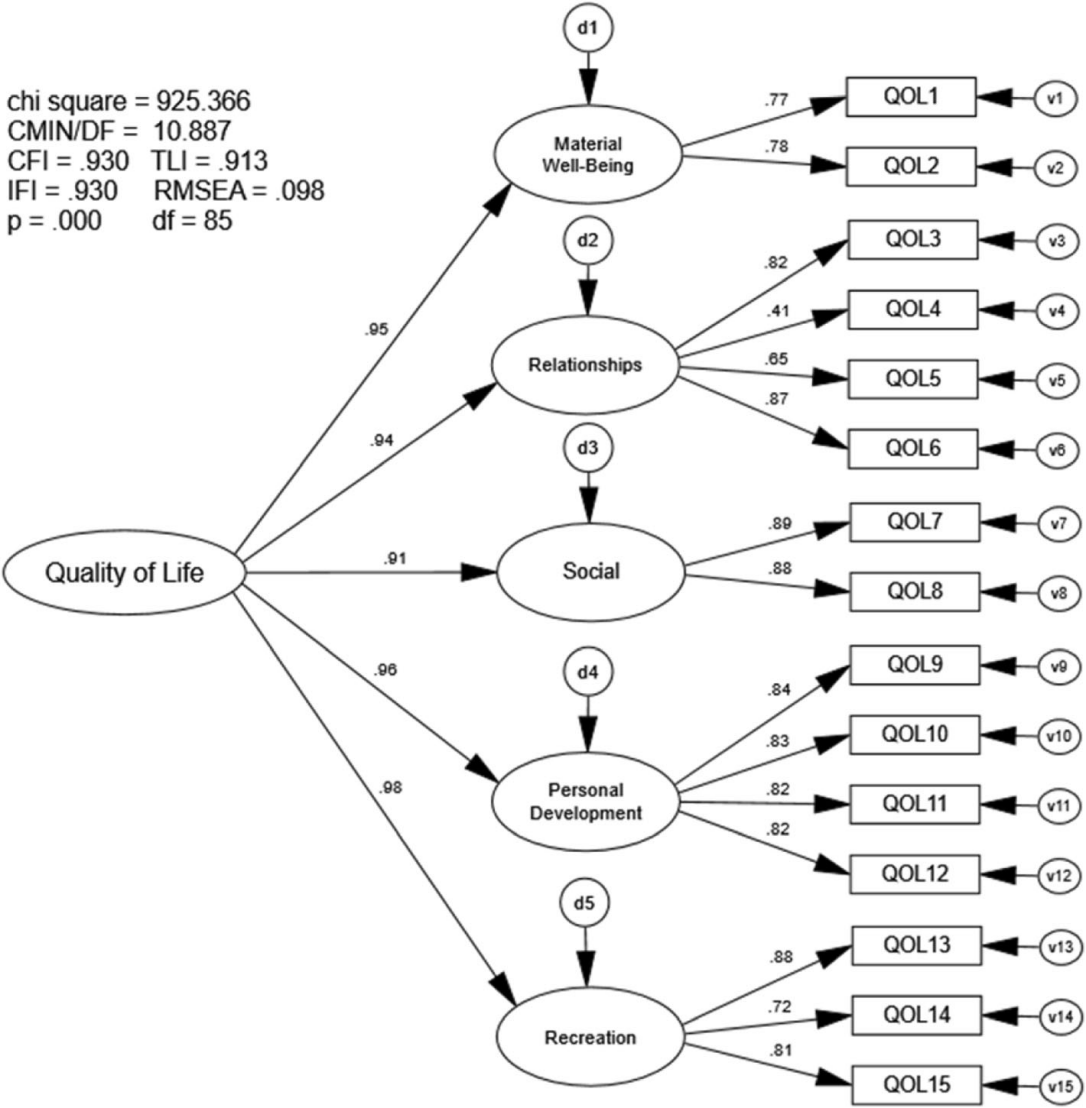
Confirmatory Factor Analysis five-factor 15-item QOLS. Chi Sq = Chi Square (χ2), CMIN/DF = the χ2 / degrees of freedom ratio; CFI = Comparative Fit Index; TLI = Tucker-Lewis Index; IFI = Bollen’s Incremental Fit Index; RMSEA = Root Mean Square Error of Approximation, df = degrees of freedom, p = alpha level

**Fig. 2:**
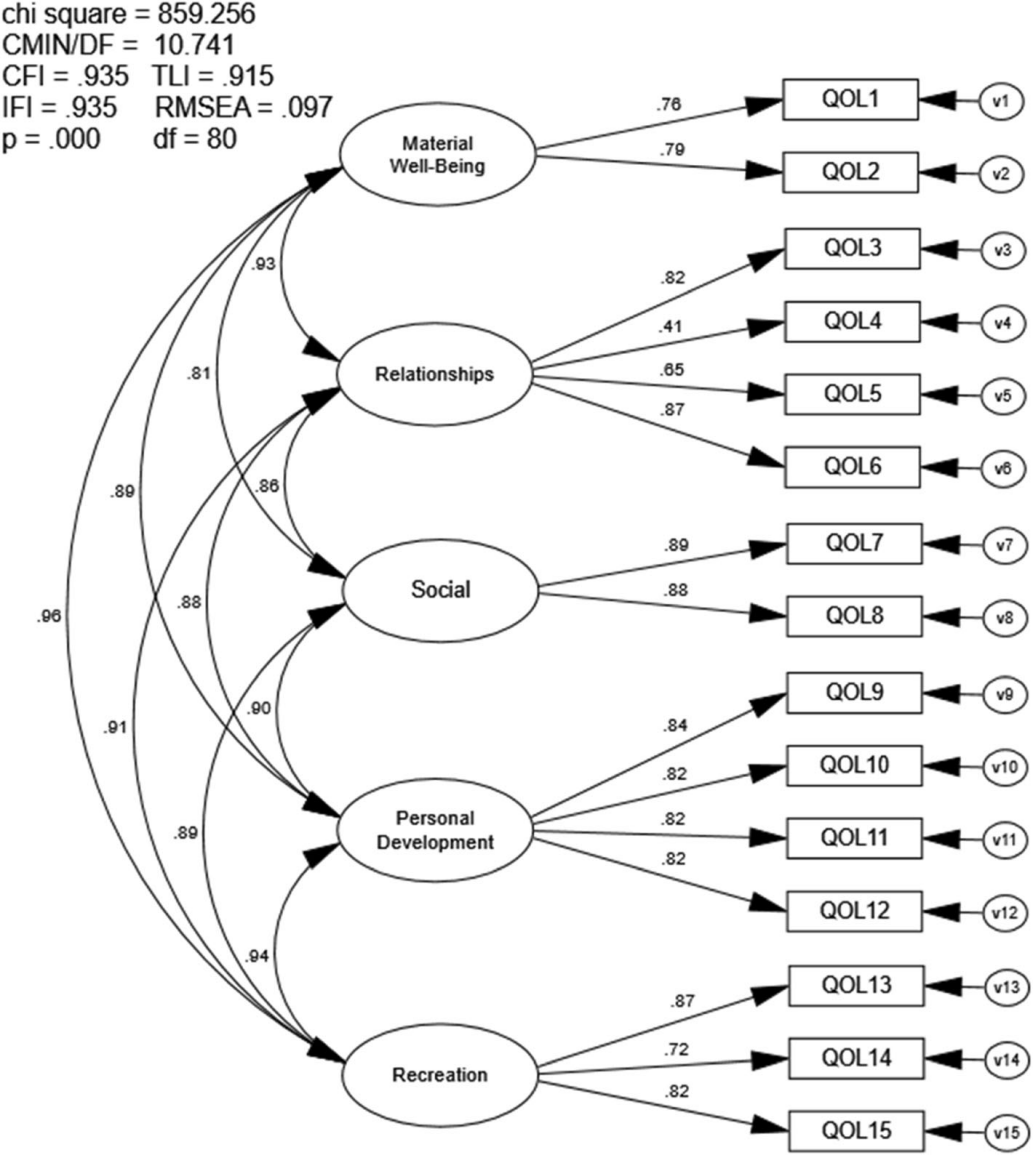
First-Order Confirmatory Factor Analysis five-factor 15-item QOLS. Chi Sq = Chi Square (χ2), CMIN/DF = the χ2 / degrees of freedom ratio; CFI = Comparative Fit Index; TLI = Tucker-Lewis Index; IFI = Bollen’s Incremental Fit Index; RMSEA = Root Mean Square Error of Approximation, df = degrees of freedom, p = alpha level

The CFA of the five-factor, 15-item QOLS indicated marginal, but not preferred model fit to the sample data. The goodness-of-fit indices approached but did not meet recommended values (CFI = .930, TLI = .913, RMSEA = .098, IFI = .930; Fig. 1). Moreover, correlations between first-order latent variables (e.g., ‘Material Well-Being, ‘Relationships’, etc.) were very high, ranging from r = .81 to r = .96 (Fig. 2).

### Confirmatory factor analysis three-factor 16-item Quality of Life Scale

**Fig. 3:**
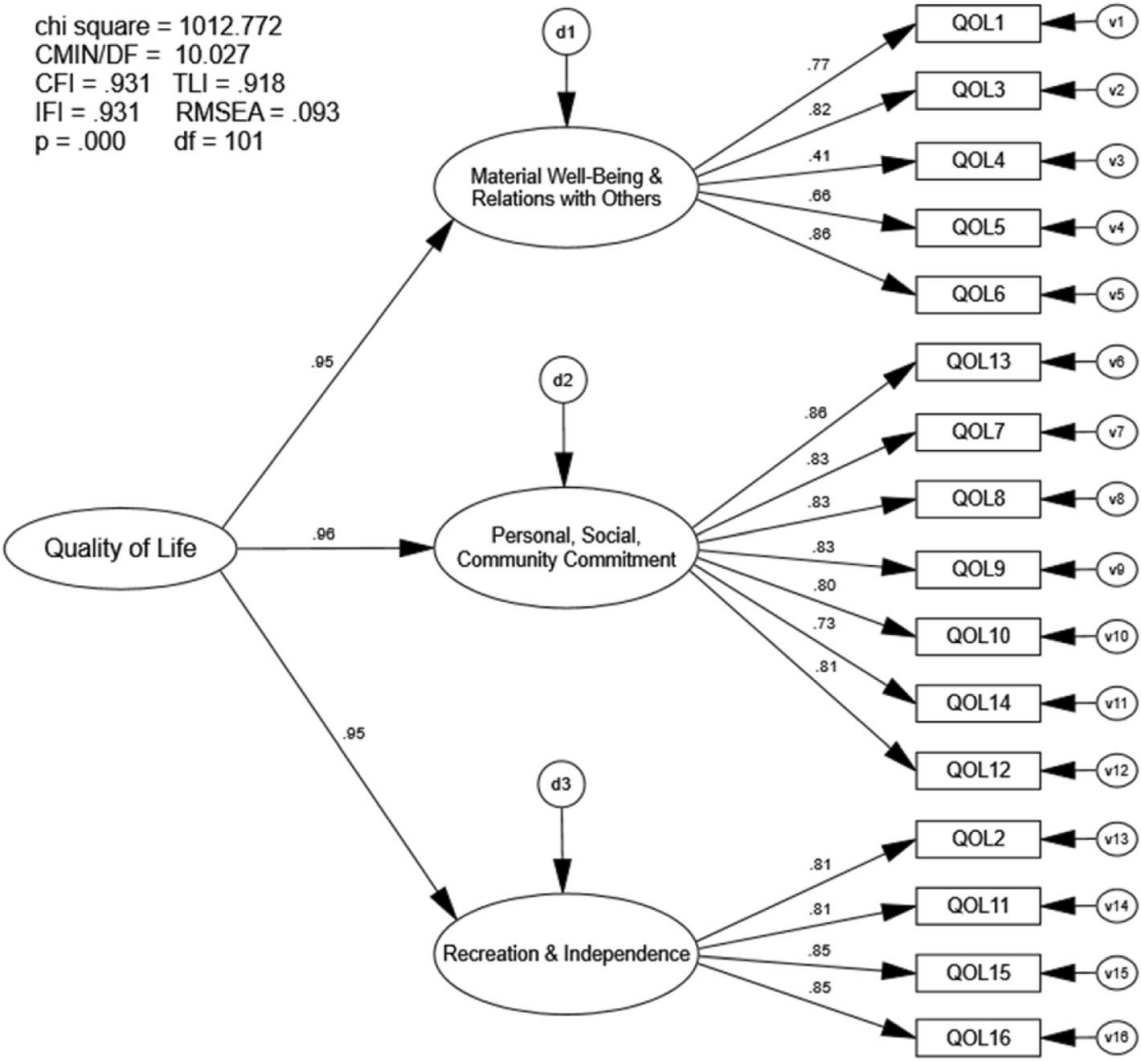
Confirmatory Factor Analysis three-factor 16-item QOLS. Chi Sq = Chi Square (χ2), CMIN/DF = the χ2 / degrees of freedom ratio; CFI = Comparative Fit Index; TLI = Tucker-Lewis Index; IFI = Bollen’s Incremental Fit Index; RMSEA = Root Mean Square Error of Approximation, df = degrees of freedom, p = alpha level

**Fig. 4:**
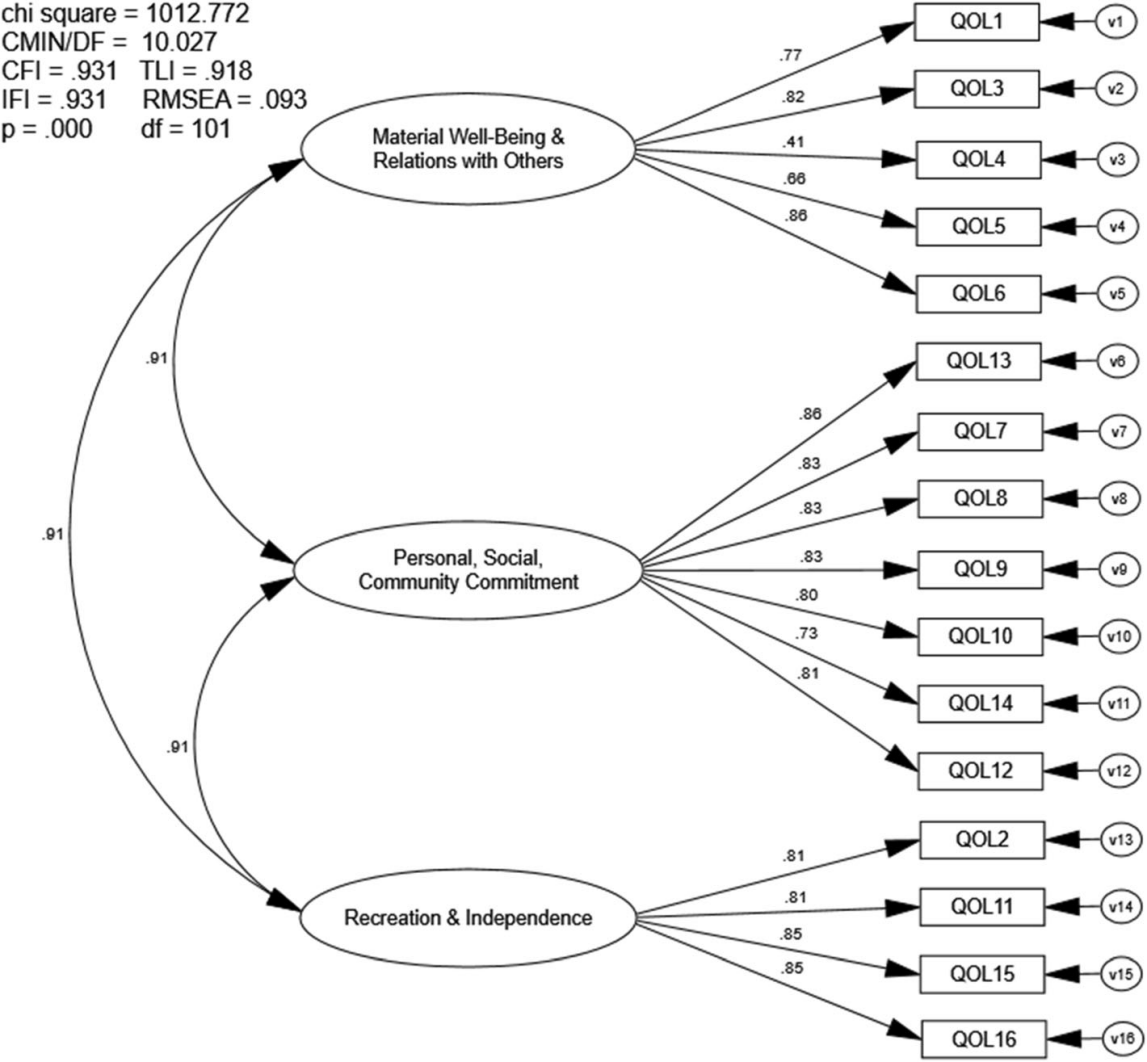
First-Order Confirmatory Factor Analysis three-factor 16-item QOLS. Chi Sq = Chi Square (χ2), CMIN/DF = the χ2 / degrees of freedom ratio; CFI = Comparative Fit Index; TLI = Tucker-Lewis Index; IFI = Bollen’s Incremental Fit Index; RMSEA = Root Mean Square Error of Approximation, df = degrees of freedom, p = alpha level

The CFA of the three-factor, 16-item QOLS also indicated marginal, not preferred model fit. The goodness-of-fit indices approached but did not meet recommended values (CFI = .931, TLI = .918, RMSEA = .093, IFI = .931; Fig. 3). Correlation values between all three first-order latent variables were high (r =.91) (Fig. 4).

### Scale structure of modified Quality of Life Scale

#### Identification of a modified Quality of Life Scale

Initial EFA of the QOLS using sample n_1_ (*n* = 518) extracted two dimensions (Table 6). Items 4, 5, and 15 were eliminated due to low loadings or high cross loadings. Items 6, 7, 9, 10, 12, 13, 14, 16 were removed due to inflated Cronbach’s alpha levels, high correlation values, or lack of conceptual relevance (i.e. rearing children) to certain groups in the population. The resulting single-factor, five-item scale consisted of items 1, 2, 3, 8, and 11 from the original 16-item QOLS. The single factor accounted for 58.9% of the variance in the five retained items, with all item loadings ≥ .75. Cronbach’s alpha and omega = .89 (Table 7). This brief version of the QOLS better satisfied a priori statistical guidelines.

**Table 6:** Initial exploratory factor analysis loadings (*n*=518)

Item	Factor 1	Factor 2
1. “Material comforts home, food, conveniences, financial security…”	**.584**	−.246
2. “Health – being physically fit and vigorous…”	.279	**−.746**
3. “Relationships with parents, siblings & other relatives – communicating, visiting, helping…”	**.710**	−.139
4. “Having and raising children…”	**.530**	.162
5. “Relationships with spouse or significant other…”	**.668**	.051
6. “Close friends…”	**.756**	−.136
7. “Helping and encouraging others, volunteering, giving advice…”	**.821**	.007
8. “Participating in organizations and public affairs…”	**.791**	−.047
9. “Learning – attending school, improving understanding, getting additional knowledge…”	**.819**	−.013
10. “Understanding yourself – knowing your assets and limitations – knowing what life is about…”	**.840**	.016
11. “Work – job or in home…”	**.773**	−.096
12. “Expressing yourself creatively…”	**.818**	.050
13. “Socializing – meeting other people, doing things, parties, etc.”	**.748**	−.176
14. “Reading, listening to music, or observing entertainment…”	**.700**	−.023
15. “Participating in active recreation…”	.461	**−.542**
16. “Independence, doing for yourself…”	**.664**	−.258
**Cronbach’s Alpha | Omega**	.95 | .95	.88 | .89

**Table 7:** New proposed QOLS model (n = 518)

Item	Factor 1
11. “Work – job or in home…”	.814
3. “Relationships with parents, siblings & other relatives – communicating, visiting, helping…”	.758
1. “Material comforts home, food, conveniences, financial security…”	.800
2. “Health – being physically fit and vigorous…”	.830
8. “Participating in organizations and public affairs…”	.750
**Cronbach’s Alpha | Omega**	.89 | .89

#### Validation analysis of the modified Quality of Life Scale

**Fig. 5:**
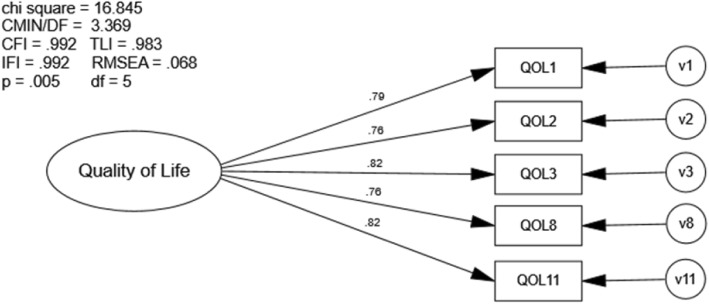
Covariance Model of Modified QOLS. Chi Sq = Chi Square (χ2), CMIN/DF = the χ2 / degrees of freedom ratio; CFI = Comparative Fit Index; TLI = Tucker-Lewis Index; IFI = Bollen’s Incremental Fit Index; RMSEA = Root Mean Square Error of Approximation, df = degrees of freedom, p = alpha level

Covariance modeling of the modified QOLS using sample n_1_ indicated good model fit (χ^2^ [5] = 16.845, *p* ≤ .005; CFI = .992; RMSEA = .068; Fig. 5). The majority of fit indices values exceeded recommended values, while RMSEA levels approached the highest recommended levels. All factor loadings were significant (*p* ≤ .001), and modification indices did not suggest model fit could be substantially improved with the specification of any non-zero covariances between error terms.

#### Confirmatory factor analysis of modified Quality of Life Scale

**Fig. 6:**
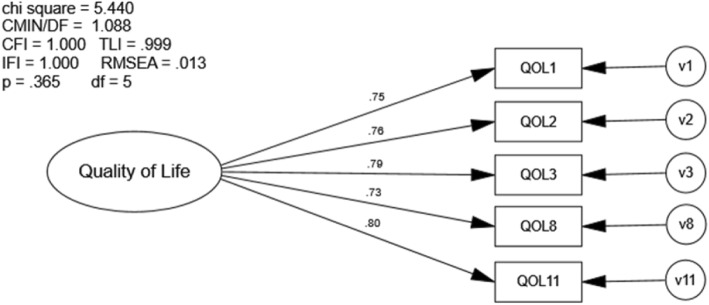
Confirmatory Factor Analysis of Modified QOLS. Chi Sq = Chi Square (χ2), CMIN/DF = the χ2 / degrees of freedom ratio; CFI = Comparative Fit Index; TLI = Tucker-Lewis Index; IFI = Bollen’s Incremental Fit Index; RMSEA = Root Mean Square Error of Approximation, df = degrees of freedom, p = alpha level

Confirmatory factor analysis using sample n_2_ also indicated very good model fit. All of the fit indices calculated exceeded recommended values (χ^2^ [5] = 5.44, *p* = .365; CFI = 1.0; RMSEA = .013; Fig. 6). All item-factor loadings were statistically significant (*p* ≤ .001) and ranged from .73 to .80.

### Invariance testing for sex subgroups

From the full sample, males (*n*=387) and females (*n*=641) were used for invariance testing. The initial configural model demonstrated very good model fit (CFI = .994; χ^2^ = 23.245; RMSEA = .036; Table 8), indicating the form of a basic five-item model structure was invariant across sex. The metric model (i.e., equal loadings) also passed both the CFI_DIFF_ test and the χ^2^_DIFF_ test. Because the five-item QOLS satisfied metric (equal loadings) invariance criteria, examining an equal latent QoL variance structure was warranted. Results indicated both the CFI_DIFF_ and χ^2^_DIFF_ non-invariant criteria were exceeded (Table 8). When variances were not constrained to be equal, the female sub-sample exhibited substantially more variability on latent QoL than did the male sub-sample (male variance = 0.47, female variance =1.46.)

**Table 8:** Goodness-of-fit indices for the measurement invariance analyses across sex

	*χ*^*2*^	df	*χ*^*2*^_diff_ (df_diff_)	CFI	CFI_diff_	TLI	RMSEA
Males (*n* = 387)	15.58	5	–	0.987	–	0.973	0.074
Females (*n* = 641)	7.65	5	–	0.998	–	0.997	0.029
Model A (equal form)	23.25	10	–	0.994	–	0.989	0.036
Model B (equal loadings)	31.45	14	8.20(4)	0.993	0.001	0.99	0.035
Model C (equal factor variances)	58.63	15	**35.39(5)**	0.982	**0.012**	0.976	0.053
Model D (equal indicator intercepts)	60.31	18	**37.07(8)**	0.982	**0.012**	0.98	0.048
Model E (equal latent means)	148.24	19	**124.99(9)**	0.946	**0.048**	0.943	0.081

The scalar model (i.e., equal loadings and intercepts) exceeded the χ^2^_DIFF_ test criteria, and just exceeded the CFI_DIFF_ test criteria (Table 8), which suggested potential item-level bias between males and females. Follow-up analysis indicated Item #2 exhibited slight bias (i.e., when Item #2 was not restricted to be equivalent across both groups, the revised five-item model then met invariance criteria).

### Invariance testing physically active-healthy and physically active-injured subgroups

From the full sample, the physically active-healthy (*n*=151) and physically active-injured (*n*=470) subgroups were used for invariance testing. The initial model (configural) demonstrated very good model fit (CFI = .989; χ^2^ = 16.702; RMSEA = .033; Table 9), indicating the basic five-item model structure was invariant across the PA-H and PA-I sub-groups. The metric model (i.e., equal loadings) also passed both the CFI_DIFF_ test and the χ^2^_DIFF_ test. The five-item QOLS metric invariance warranted testing of equal latent QoL variance. Both CFI_DIFF_ and χ^2^_DIFF_ criteria were met (Table 9). Thus, both PA-H and PA-I sub-samples exhibited similar variability on the latent QOLS dimension.

**Table 9:** Goodness-of-fit indices for the measurement invariance analyses across physically active-healthy and physically active-injured subgroups

	*χ*^*2*^	df	*χ*^*2*^_diff_ (df_diff_)	CFI	CFI_diff_	TLI	RMSEA
PA-H (*n* = 151)	9.87	5	–	0.973	–	0.947	0.081
PA-I (*n* = 470)	6.80	5	–	0.996	–	0.991	0.028
Model A (equal form)	16.70	10	–	0.989	–	0.977	0.033
Model B (equal loadings)	21.31	14	4.61(4)	0.988	< 0.001	0.982	0.029
Model C (equal factor variances)	21.55	15	4.84(5)	0.989	< 0.001	0.985	0.027
Model D (equal indicator intercepts)	42.36	18	**25.66(8)**	0.959	**0.03**	0.954	0.047
Model E (equal latent means)	44.37	19	**27.67(9)**	0.957	**0.032**	0.955	0.046

The scalar model (i.e., equal loadings and intercepts) did not pass the CFI_DIFF_ test or the χ^2^_DIFF_ test, suggesting item-level bias (Table 9). Follow-up analysis indicated Item #2 exhibited substantial bias (i.e., when Item #2 was not restricted to be equivalent across both groups, the revised five-item model met all measurement invariance criteria for these sub-groups).

### Invariance testing for physically active-healthy and osteoarthritis subgroups

From the full sample, the physically active-healthy (*n*=151) and osteoarthritis (*n*=131) subgroups were used for invariance testing. The initial model (configural) demonstrated very good model fit (CFI = .986; χ^2^ = 15.941; RMSEA = .046; Table 10), indicating equal form of the five-item model for both groups. The metric model (i.e., equal loadings) passed both the CFI_DIFF_ test and the χ^2^_DIFF_ test. Because the five-item QOLS satisfied metric model invariance criteria, an equal latent QoL variance model was warranted. Both CFI_DIFF_ and χ^2^_DIFF_ non-invariant criteria were exceeded (Table 10). When variances were not constrained to be equal, the OA sub-sample exhibited substantially more variability on latent QoL than did PA-H group (PA-H variance = 0.51, OA variance =1.40.)

**Table 10:** Goodness-of-fit indices for the measurement invariance analyses across physically active-healthy and osteoarthritis subgroups

	*χ*^*2*^	df	*χ*^*2*^_diff_ (df_diff_)	CFI	CFI_diff_	TLI	RMSEA
PA-H (n = 151)	9.87	5	–	0.973	–	0.947	0.051
OA (n = 131)	6.07	5	–	0.996	–	0.991	0.041
Model A (equal form)	15.94	10	–	0.986	–	0.972	0.046
Model B (equal loadings)	18.83	14	2.89	0.989	< 0.001	0.984	0.035
Model C (equal factor variances)	42.63	15	**26.69**	0.936	**0.05**	0.914	0.081
Model D (equal indicator intercepts)	101.30	18	**85.36**	0.806	**0.18**	0.784	0.129
Model E (equal latent means)	216.57	19	**200.63**	0.54	**0.446**	0.515	0.193

The scalar model (i.e., equal loadings and intercepts) did not pass the CFI_DIFF_ test or the χ^2^_DIFF_ test, again suggesting item-level bias between health status subgroups (Table 10). When Item #2 was not restricted to be equivalent across both groups, the revised five-item model met all measurement invariance criteria.

### Follow-up analysis on a proposed four-item QOLS

Because the second item of the revised five-item QOLS was a consistent source of non-invariance and item-level bias for all subgroup analyses, invariance procedures were repeated after eliminating this item. Results are displayed in Table 11. In summary, a four-item version exhibited measurement invariance for all conditions and subgroups, except for the scalar invariance model when comparing PA-H individuals to the OA sub-sample. For this comparison, Item #3 exhibited biased responses.

**Table 11:** Goodness-of-fit indices for measurement invariance analyses - four-item model across sex and health status subgroups

	*χ*^*2*^	df	*χ*^*2*^_diff_ (df_diff_)	CFI	CFI_diff_	TLI	RMSEA
Males (*n* = 387)	4.31	2	–	0.996	–	0.988	0.055
Females (*n* = 641)	5.13	2	–	0.997	–	0.992	0.049
Model A (equal form)	9.44	4	–	0.997	–	0.991	0.036
Model B (equal loadings)	10.54	7	1.10(3)	0.998	< 0.001	0.996	0.022
Model C (equal factor variances)	41.57	8	**32.13(4)**	0.981	**0.016**	0.971	0.064
Model D (equal indicator intercepts)	14.41	10	4.97(6)	0.997	< 0.001	0.997	0.021
Model E (equal latent means)	86.83	11	**77.39(7)**	0.956	**0.041**	0.952	0.082
PA-H (*n* = 151)	7.30	2	–	0.965	–	0.895	0.133
PA-I (*n* = 470)	2.83	2	–	0.997	–	0.992	0.03
Model A (equal form)	10.16	4	–	0.987	–	0.96	0.05
Model B (equal loadings)	12.93	7	2.78(3)	0.987	< 0.001	0.978	0.037
Model C (equal factor variances)	13.49	8	3.34(4)	0.988	0.001	0.982	0.033
Model D (equal indicator intercepts)	14.59	10	4.43(6)	0.99	0.003	0.98	0.027
Model E (equal latent means)	15.03	11	4.88(7)	0.991	0.004	0.99	0.024
PA-H (*n* = 151)	7.30	2	–	0.965	–	0.895	0.133
OA (*n* = 131)	1.43	2	–	1	–	1.01	0
Model A (equal form)	8.73	4	–	0.986	–	0.957	0.065
Model B (equal loadings)	11.29	7	2.56(3)	0.987	0.001	0.978	0.047
Model C (equal factor variances)	34.33	8	**25.60(4)**	0.921	**0.065**	0.881	0.108
Model D (equal indicator intercepts)	24.16	10	15.43(6)	0.957	**0.029**	0.949	0.071
Model E (equal latent means)	105.22	11	**96.50(7)**	0.717	**0.269**	0.691	0.175

As with the five-item scale, females reported higher levels of variability than did males when latent QoL was based on the four-item scale. The invariant scalar model results warranted comparison of reported levels of QoL between males and females. Based on the four-item QOLS, females reported higher levels of QoL than did males. Likewise, consistent with the five-item scale, the four-item QOLS exhibited no difference in variability on latent QoL scores when PA-H individuals were compared to the PA-I sample. Further, there was not any apparent difference of average levels of QoL when these samples were compared using the four-item scale. Again, consistent with the five-item QOLS results, the OA sub-sample exhibited substantially more variability than did the PA-H sub-sample. The non-invariant scalar results precluded comparison of mean levels of QoL between these samples.

### Correlation analyses

The five-item QOLS was highly correlated (covariance latent variable model r = 1.0, R^2^ = 1.0; bivariate cumulative score r = .96, R^2^ = .92) with the 16-item QOLS. The four-item QOLS was also highly correlated (covariance latent variable model r = 1.0, R^2^ = 1.0; bivariate cumulative score r = .95, R^2^ = .90) with the 16-item QOLS.

## Discussion

In the present study, we aimed to identify if the proposed factor structure of previously published QOLS versions were psychometrically sound using contemporary CFA and structural equation modeling procedures in a large, heterogeneous sample. The CFA approach was used to more rigorously examine the QOLS for use in clinical practice and research [16]. We also used EFA to identify an alternative, more parsimonious structure for the QOLS. The modified QOLS was further evaluated using CFA and CFA-based invariance testing to determine if the more parsimonious QOLS measurement model better met psychometric measurement recommendations. The findings of our study suggest the original QOLS versions do not meet recommended measurement properties, and thus, challenge the appropriateness of using the QOLS as a valid multidimensional QoL assessment tool.

### Confirmatory factor analysis of the Quality of Life Scale

Prior claims of validity of the QOLS [10–15] are not supported by the inconsistent factor content reported in previously published literature. Furthermore, neither the five-factor structure nor the three-factor structure met recommended CFA psychometric properties in this study. For example, high correlation values between latent variables in both measurement models suggest the presence of substantial multicollinearity among the claimed distinct dimensions [17, 19]. These characteristics, combined with inadequate overall model fit of the CFAs and potential multicollinearity of the proposed sub-dimensions (i.e., high latent variable correlations), contradict previously assumed validity of the multidimensionality of the QOLS [10–15]. Without a psychometrically sound measurement model (either 15- or 16-item version), there was no justification for pursing the invariance analyses of the original QOLS scales. However, our results did warrant a specification search for a more psychometrically desirable solution using QOLS items [19].

### Psychometric analysis of a modified Quality of Life Scale

A single factor, five item solution, representing overall QoL, emerged from our analysis. The modified scale included at least one item from four of the five originally proposed factors (i.e., PMWB, REL, SCC, PDF) in the 15-item version, but no items from the original ‘Recreation’ factor. Of the originally proposed three-factor, 16-item scale, the new version included at least one item from each factor (i.e., RMW = 2, HF = 2, PSCC = 1). Although all five originally proposed factors were not represented in the modified scale [10], it still comprised a wide variety of items that represented different aspects of the theorized construct of QoL [10].

The new five-item QOLS was then subjected to confirmatory analysis. Statistically, the new five-item scale exceeded a-priori guidelines for model fit [17], offering encouraging results for the possibility of using five items to adequately measure overall QoL. The summative scores on the new five-item scale and original 16-item scale were highly correlated (r = .96), indicating that most of the variance (R^2^ = .92) in participant responses from the 16-item scale was accounted for using only five items. This finding reiterates the item redundancy issues observed in the original model, and further suggests that the included five items assess the proposed QoL construct as well as all sixteen items.

Unfortunately, follow-up invariance testing of the modified QOLS by sub-groups (i.e., sex and health status) produced mixed results. As evidenced by the configural invariance models, the basic five-item structure did hold up in form for the sub-groups examined. Furthermore, the metric invariance models demonstrated that subgroups exhibited a consistent covariance structure among the five items. These results provide support for potentially using the five-item QOLS version to examine relationships of QoL with other constructs [17]. However, the five-item scalar measurement models failed to provide evidence supporting valid use of the new scale to compare subgroup levels (i.e. “amounts”) of QoL. The prime contributor to this measurement bias appeared to be Item #2, which taps into physical health status. Upon reflection, these results are not surprising given that two of the three subgroup analyses examined were comparisons of physically active healthy individuals to those with a physical injury or physical activity limiting condition.

Reducing the scale even further by removing the problematic Item #2 resulted in a more psychometrically sound scale that appears to measure a consistent construct for some of the subgroups tested. However, the further abbreviated four-item version still failed the scalar invariance test for comparing the PA-H group to the OA group. Thus, use of this scale would only be appropriate for examining differences in relationships of QoL with other constructs without comparing actual levels of QoL for certain subgroups. Further, it can be argued that removing the only indicator representing physical health might represent a meaningful alteration of what underlying construct is being assessed in groups suffering from a pathology affecting physical health.

### Implementation in clinical practice and research

Assessing patient reported QoL is an important component of healthcare research and practice; however, we do not recommend assessment and interpretation of QoL using the 15- or 16-item QOLS versions. Examining the items beyond the statistical analysis of the scale reveals inherent design flaws that we believe contributed to the poor psychometric properties of the scale. In particular, concerns arose regarding redundant, double-barreled (i.e., asking about two or more ideas at once) items and whether the response Likert scale consistently matched question structure. Double-barreled questions are problematic because a respondent does not know which part of the item to respond to when selecting their Likert score. Thus, the use of double-barred question causes confusion and inconsistent responses among participants, which results in subsequent analysis complications [26]. When examining the original QOLS items [10], we noted that many questions were double-barreled or more extreme (e.g., lists of several activities, etc.) [26].

Further, the Likert scale used for the QOLS is bipolar (i.e., has a negative and positive end) which potentially creates multiple problems for participant interpretation. First, the endpoints are “terrible” and “delighted,” and these descriptors may not be seen as “opposites,” which is recommended when using bipolar scales [26]. Second, the 1–7 scale does not have a neutral point, even though the “terrible” to “delighted” scale theoretically does [26]. Third, the verbiage of the scale options (i.e., “terrible” to “delighted”) does not match the instructions given or follow an expected sequential order for respondents [26]. A more effective Likert scale, following contemporary survey recommendations may be one ranging from − 3 to + 3 that included similar wording on either end [26]. The item and Likert scale design issues may explain in part why the factor structure was so inconsistent across multiple samples in the literature [10–15], as well as the present study.

Inherent design flaws, as well as the concerns identified during CFA, indicate the original QOLS are not fit for use in clinical practice or research in their current form. The modified scales met initial testing standards, but the invariance testing results indicate caution is warranted when using the scales. At minimum, researchers and clinicians should be careful when interpreting group comparisons of QoL between subgroups in any investigation using these QOLS items as indicators of QoL. Because the evidence does not suggest the original or modified QOLS versions meet all contemporary recommendations (e.g., CFA fit indices recommendations, invariance testing recommendations, etc.), it would be imprudent to recommend the scale to accurately measure QoL, or changes in patient-perceived QoL, across various populations. Instead, we recommend either: 1) developing a new instrument to adequately assess all aspects of QoL, 2) choosing another existing QoL instrument and performing the necessary analysis to establish the psychometric properties of the scale meet current recommendations, or 3) identify an instrument that has met CFA and invariance guidelines and is ready for implementation in research and clinical practice.

### Limitations and future research

While the present study has confirmed the lack of factorial validity of the QOLS, there are still limitations to consider. The five-item modified QOLS EFA and covariance model was assessed with a cross-validation sample to confirm the proposed model held in a new sample. However, the responses used for the cross-validation procedures were from a sample of participants who responded to all 16 items of the QOLS. Thus, it is possible that the responses to the five items were influenced by the other items not included in the final model. Therefore, further testing is needed to confirm the model fit of the modified QOLS when participants are only provided with those five items in the scale. Further, while we had a large and diverse sample, we did not conduct long-term follow-up or compare results with another criterion scale. Because of the study design, we could not perform test-retest reliability, perform longitudinal invariance testing, or establish scale responsiveness.

Assessing QoL is a vital component of providing quality patient care. Therefore, future research should aim to define QoL in a concise and universal manner, as the inconsistency of this definition appears to be one of the major obstacles in developing an adequate instrument. After a definition has been established, future research should identify or create an instrument that is psychometrically sound and can be used effectively in research and clinical practice. Finally, researchers should collect longitudinal data in diverse populations (e.g., pediatric, geriatric, injured, healthy, physically active, sedentary, etc.) to allow for the completion of all necessary analyses to establish scale reliability and validity.

## Conclusions

The proposed construct validity of 15- and 16-item multidimensional QOLS versions was not substantiated by the findings in our study. Although our analyses identified a modified-QOLS that appeared to be a more psychometrically sound instrument, the modified version exhibited bias at the item level. The modified QOLS might be useful for addressing a limited set of associative research questions within certain sub-group populations. However, given its inconsistent psychometric properties across all sub-groups, combined with potential item design flaws and incomplete psychometric testing, we cannot recommend the modified version for widespread use by clinicians or researchers at this time. The need to measure QoL remains an important concept in healthcare, but improved assessment tools validated using contemporary technique are necessary to ensure the instrument is valid for use with various patient populations and subgroups.

## Data Availability

Datasets used and analyzed are available from the corresponding author upon reasonable request.

## Abbreviations

ADL: Activities of daily living
CL: Cross-loading (i.e., ≥ .30 but <.40)
CL-E: Cross-loading, extreme (≥.45)
DNF: Did not factor (i.e., all loadings <.30)
DNL: Did not load (i.e., all loadings <.40 but >.30)
HF: Health and functioning
MSK-C: Musculoskeletal Pathology with a Comorbidity
NI: Item not included in analysis
OA: Osteoarthritis
PA-H: Physically-Active Healthy
PA-I: Physically-Active Injured
PSCC: Personal, social, and community commitment
QoL: Quality of Life
QOLS: Quality of Life Scale
RMW: Relationships and material well-being
